# Assessing the Quality of the Endocervical Component: Pitfalls to Cervical Cancer Screening in Limpopo Province, South Africa

**DOI:** 10.1177/10732748251363746

**Published:** 2025-08-13

**Authors:** Dorah Ursula Ramathuba, Doris Ngambi

**Affiliations:** 1 Department of Advanced Nursing Science, 56868 University of Venda, Thohoyandou, South Africa

**Keywords:** assessment, cervical cancer, cervix, Pap smear, specimen adequacy

## Abstract

**Background:**

Specimen adequacy is an essential indicator of screening programme performance. The effectiveness and efficiency of Pap tests are classified in the laboratory based on their adequacy for interpretation as satisfactory or unsatisfactory.

**Purpose:**

The purpose of the study was to determine the processes of collecting, storing, transporting, and evaluating Pap smears in rural health facilities in Limpopo Province, South Africa.

**Method:**

A mixed-method research approach was used for the study. An exploratory sequential mixed-methods design was employed to collect and analyse qualitative data, and then use the findings to develop an instrument in a subsequent quantitative phase, thereby increasing the breadth and depth of understanding of the phenomena under study. The population comprised professional nurses, operational managers, and laboratory technicians. The qualitative strand explored challenges to cervical cancer screening, while the quantitative strand described factors contributing to the inadequacy of the cervical component. The study was conducted from July 2019 to February 2020. The results were merged for triangulation.

**Findings:**

The inadequacy rates reported by districts ranged between 38% and 50%. The findings revealed that professional nurses lacked adequate knowledge of the skills required for collecting, labelling, and storing Pap smears before dispatch. Furthermore, the in-service training provided was poorly coordinated and unstructured, and other professional nurses were not keen on screening for cervical cancer, resulting in poor health outcomes for women in the community who had to return for repeat smears.

**Conclusion:**

Inadequacy of the transformation zone component and unsatisfactory smears have a higher risk of progression to cervical cancer or pre-cancer lesion than adequacy of the transformation zone.

## Introduction

According to the World Health Organization (WHO),^1^ cervical cancer is the fourth most common cancer in women globally, with around 660 000 new cases and around 350 000 deaths in 2022. Countries around the world are working to accelerate the elimination of cervical cancer by 2030. Worldwide, cervical cancer rates have decreased dramatically in developed countries due to the increase in screening efforts. However, the incidence and prevalence in developing countries remain high due to uncoordinated or a lack of screening programmes, with approximately 80% of all cervical cancer deaths occurring in the developing world.^1^ The WHO Global Strategy defines elimination as reducing the number of new cases annually to 4 or fewer per 100 000 women, which can only be achieved through adequate assessment.^2^

Cytology-based screening programmes for cervical cancer have been effective in reducing cancer incidence and preventing premature deaths worldwide. Cytopathology techniques, such as the Pap smear, have played a significant role in cancer prevention. The quality of collecting and evaluating cervical cytology or histology specimens plays a significant role in the early detection of cervical abnormalities. It serves as the basis for further clinical management and the improvement of health outcomes.^3,4^ High-quality screening with cytology (Papanicolaou test) has significantly reduced mortality from squamous cell cervical cancer (sc-K), which comprises 80% to 90% of cervical cancers.^4^

In South Africa, cervical cytology can be taken by registered nurses and midwives, usually in Primary Health Care (PHC) facilities. Some are trained on the job and with continuous in-service programmes by the reproductive health coordinators in the various districts. Mills et al^5^ indicate that practice nurses taking Pap smears should have undertaken a credited training course, and continuing professional development must be part of the credentialing arrangement for all nurses. Parra et al^6^ allude that increasing medical providers’ knowledge and skills is vital for early detection and prevention of cervical cancer and cancer in medically underserved areas.

The competency and experience of the person taking the smear are crucial in obtaining smears with an adequate cellular composition from the entire transformation zone. However, Limpopo Province does not have a coordinated training programme for cervical cancer screening, and the National Health Laboratories are stationed within district hospitals, where they do not perform cytology testing. Cytology specimens are sent to the provincial laboratory for assessment. These smears may take weeks to be processed due to a lack of specialised staff or insufficient quality control measures.

The study intended to address the gaps regarding the competency of smear takers, laboratory technicians, and cytopathologists in preparing, collecting, storing, transporting, testing, and releasing results in Limpopo Province. High-quality results depend on the quality of the smears collected, which is best achieved through effective collaboration among stakeholders (professional nurses, district coordinators, and laboratory technologists). Coordination of processes and adherence to standard operating procedures are essential to ensure adequate smear quality for the early detection of precancerous and cancerous lesions, thereby facilitating timely treatment and appropriate clinical management based on diagnostic findings. Mohammedsaleh^7^ indicates that the correct procedure for specimen rejection should be applied, and the rejected sample should be accompanied by a rejection report that explains why the sample was rejected. There is a lack of process flow in Limpopo Province, and it takes a long time to obtain feedback or results, making it challenging to track clients for repeat smears if required. This is because women in rural settings are often hesitant, as they view the procedure as humiliating.

The Bethesda System (TBS) stands out as a model of standardised reporting in cervicovaginal cytology. It suggests that if the specimen is inadequate, a description provides quality indicators such as the presence or absence of an endocervical or transformation zone component, partially obscuring blood, inflammation, etc.^3^ Designating smears as unsatisfactory alerts clinicians that the smear may not be reliable for detecting pre-neoplastic or neoplastic conditions.^8^ Such reports are rarely received from our laboratories, which hampers the control and anticipated eradication of cervical cancer by 2030, as set in the South African Strategic National Framework.^9^

## Problem Statement

In Limpopo Province, the districts were unable to reach the 70% adequacy rate, as indicated by the National Health Laboratory Services reports during quarterly review meetings for 2016-2018. These meetings reviewed and discussed the performance of all facilities under Vhembe and Mopani Districts. Most of the Pap smears collected were of poor quality, with no endocervical component, or were unsatisfactory for evaluation, as confirmed by Pap smear results from most facilities within the 2 districts. High inadequacy has a significant association with cost, as women need to be re-screened in the event of an inadequate specimen, and many women may have been issued with false negative results, thus reducing the benefits of screening for cervical cancer.

Mulongo et al^10^ indicate that the median adequacy rate, which measures whether there is sufficient endocervical tissue for analysis, is 47%, and many ‘inadequate’ smears must be repeated, resulting in unnecessary costs and attrition from care. Furthermore, the authors contend that the low adequacy rate may be attributed to the choice of sample collection device, specifically the use of a wooden Ayre spatula, which is associated with suboptimal sampling of the endocervix and a higher rate of inadequate smears compared to the use of a cytobroom.^10,11^ High numbers of inadequately/unsatisfactorily performed smears impact negatively on the quality of life for women as they delay early detection for cervical cancer and treatment. The adequacy rate could be increased at the health facility level through additional staff training on accurately collecting cervical cells.^12^

## Methods

The study employed an exploratory sequential mixed-methods design, a design in which the researcher first explored qualitative data and analysed it, and then used the findings to develop an instrument in a second quantitative phase, thereby combining qualitative and quantitative research methods into a single research study.^13^ It involves collecting and analysing qualitative and quantitative data to better understand a phenomenon and answer the research questions. A convergent design was validated and illustrated, with both qualitative findings and quantitative results, and conclusions drawn, consolidated, and interpreted.^13^ The purpose of the exploratory sequential design was to provide a broader understanding of the complex phenomena of cervical cancer screening, generating content for the questionnaire, thereby offering readers more assurance in research findings and conclusions. Phase 1a of the qualitative exploratory study helped expose a relatively unknown research area, providing insight into how the Pap smear technique was practised within the cervical cancer screening programme. According to de Vos et al,^14^ the exploratory design enabled the researcher to collect in-depth information from health care professionals, who narrated their practice competence regarding the collection of Pap smears for cytology examination. Phase 1b employed a non-experimental, cross-sectional, descriptive design with a quantitative approach to assess the knowledge, skills, and practices of health professionals regarding cervical cancer screening. Concepts related to cervical cancer screening were described and discussed using an exploratory, descriptive design, and relationships were noted. Qualitative and quantitative results were compared, and points of contention and areas of convergence were identified in the final analysis phase to form meta-interferences, thereby developing an overall understanding through integrating data strands. This approach led to a more comprehensive understanding of a research problem.

### Population

The target population included professional nurses in PHC facilities and laboratory technicians in the province. For the qualitative exploratory and descriptive design, the sample consisted of 18 professional nurses, including 2 males, 2 laboratory technicians, and 16 females. For the quantitative design, the sample size consisted of 130 health professionals, comprising 111 females and 19 males. The inclusion criteria were professional nurses working at the PHC facility for more than a year and involved in Pap smear collection, as well as laboratory technicians working at National Health Laboratories in the province, who were engaged in cytology screenings. Professional nurses with less than a year of experience in PHC were excluded.

Their professional experience ranged from 5 to 15 years. Most had bachelor’s degrees in nursing, only a few had Diplomas in Nursing and Midwifery, and the laboratory technicians were qualified medical technologists. Convenience sampling, a form of non-probability sampling, was employed because it involves using the most readily available health care professionals as study participants. Primary health care facilities were also purposively sampled; only those with the highest inadequacy rates for cervical cancer in the selected municipalities were sampled.^15^ For the quantitative approach, a total of 73 PHC facilities were identified, and systematic sampling was applied per district, resulting in a sample size of 22.

### Data Collection and Analysis

The study consisted of a face-to-face, unstructured interview and a cross-sectional survey conducted from July 2019 to February 2020. In the first phase, 1a of the qualitative exploratory design, participants described their experiences when undergoing cervical cancer screening. The researcher used bracketing during the interviews with participants to prevent research bias. The interviews were audiotaped, and field notes were recorded. The following central question was asked: *“Can you please describe your challenges regarding cervical cancer screening practice?”* The interviews were conducted in private rooms to ensure privacy and confidentiality, and lasted approximately an hour. The researcher initiated communication to break the ice, relaxed each participant, and encouraged open communication by employing effective communication skills, such as nodding, asking questions, and providing clarifications, to facilitate discussion until no new information was available. Data were analysed using the Tesch method of qualitative analysis, where an audiotape was listened to and the data transcribed. Data were read and interpreted; an independent coder assisted with coding, and a consensus was finally reached to ensure the trustworthiness. Dependability and confirmability were achieved by providing a detailed description of the data and methodology.^16^

A quantitative cross-sectional survey 1(b) was conducted using a structured questionnaire with 130 professional nurses. The questionnaire consisted of 4 sections: biographical information, knowledge, skills, and practice.^17^ The questionnaire was designed based on a literature review, and the items from these sections were measured using a Likert scale, as well as biographical data on a nominal and ordinal scale. Before data collection, the questionnaire was pre-tested to ensure content and construct validity, ensuring that the instrument accurately measured the intended variables. The crucial areas of cervical cancer screening were identified.^17^ Pre-testing was conducted with 5 participants from municipalities that were not sampled in the district, and the concept of Pap smear and cervical cytology test was refined to ensure consistency with the same idea. The data collection took 3 months, as self-report questionnaires were distributed to the various PHC facilities and left with participants for completion. The questionnaires were collected after a week at each facility. Data were coded, and descriptive and analytic statistics were used to analyse the results using SPSS 23. A chi-square analysis was conducted to examine the associations and trends between demographic and other variables and knowledge, attitudes, and skills regarding cervical cancer screening.

Creswell and Clark^18^ indicate that integration can occur at multiple levels of a study, including the design level, methods level, or interpretation level, and in various ways, such as connecting, building, merging, or embedding. For this study, the first linking of data occurred at the design level, utilising a sequential design, where the results from the first phase of the research were used to inform the development of the second stage of the research design. Integrating individual interviews and the self-report survey data enriched the conceptualisation of the challenges experienced in providing cervical cancer screening programmes, adding to the interpretation of the investigated phenomenon. This form of data analysis, synthesis, and triangulation enhanced the trustworthiness of the findings. There was a limitation in integrating and interpreting conflicting data, particularly in the interpretation of qualitative results, where participants indicated a lack of knowledge in performing a Pap smear but quantitatively indicated competency in practising the skill, making it difficult to draw a conclusion. However, the design provided the opportunity to identify complementarity, convergence, and/or divergence. The reporting of this study conforms to GRAMMS guidelines, Standard Reporting of Qualitative Research (SRQR), and the Consolidated criteria for reporting qualitative research (COREQ) and Strengthening the Reporting of Observational Studies in Epidemiology (STROBE guidelines.^19–21^

### Ethical Considerations

All research involving humans must adhere to ethical principles. The participants were informed about the study, its purpose, and the methods required for them to give informed consent. Those who agreed to participate signed a written consent form. They were assured that participation is voluntary, that they can withdraw from the study at any time, and that the information acquired will not be used against them.^22^ Anonymity was maintained by numbering questionnaires and assigning codes to the interview transcripts. Confidentiality was ensured by storing data in a locked cupboard and encrypted passwords in the computer. Ethical clearance to conduct the study was obtained from the University Ethics Committee (SHS/19/PDC/08/1305) in May 2019, and approval to access PHC facilities was granted by the Limpopo Department of Health.

## Presentation of Findings

The theme of practice competence regarding the cervical cancer screening programme, along with its categories and sub-categories, is displayed in the table and will be discussed using quantitative data and a literature review (Table 1).

**Table 1.** Summary of Themes, Categories, and Sub-Categories

Theme	Category	Sub-category
Practice competence regarding the cervical cancer screening programme	1. Incompetency in cervical cancer practice and performance of pap smear	1.1. Results of inadequate cervical component
		1.2. Poor filling/completion of cytology form
		1.3. Lack of skill in identifying the transformation zone
		1.4. Improper technique of drying the slide
		1.5. Failure to meet the set target for the national 70% adequacy rate

Source: Ngambi D. Development of a training program to strengthen cervical cancer screening in Limpopo province, South Africa (unpublished thesis) University of Venda, 2021.

### Category 1: Incompetency in Cervical Cancer Practice and Performance of Pap Smear

Lack of practice and professional incompetence, characterised by inadequate knowledge and skills in providing excellent and quality cervical cancer screening services, was identified as a diverse challenge.

#### Sub-Category 1.1: Results of Inadequate Cervical Component

Health care professionals indicated that they are insufficiently trained to collect adequate quality smears for Pap smears. Participants express a need for sufficient knowledge about providing cervical cancer screening services.

The following are quotations from the health care professionals in the interviews:

The participant indicated, *“The cervical cancer screening results come back being inadequate, proving the incompetence of health care professionals in performing cervical cancer screening.”*

She added, *“They are still using their method, they are still using the KY jelly which is not allowed, ee…..h! You can’t use the lubricant when you are collecting the endocervical component because it is also like the jelly; there, when it goes to the lab, they will see the jelly, but they won’t know the component, meaning that the specimen has to come back.”* [N10, Female, 55 years]

Another shared, “*I have discovered other people are still using the rubbing method…the circling method, which was cancelled long ago. That is why we will be having that inadequacy and the other thing…this thing of putting the slide under the sun, I do not go for that because it is going to stay there for three to four days; what do you expect it is going to come back being inadequate because the sun is too much to this slide than to put it on the cooler a place where it is going to be dry not being interfered*.*”* [N8, Female, 45 years]

**Figure 1.**
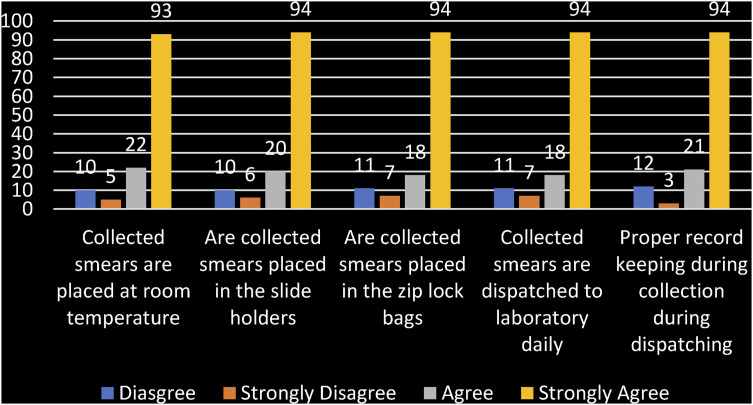
Storage and Dispatch of Smears

The quantitative results revealed that professional nurses were knowledgeable about obtaining an adequate endocervical component, with the majority (82.3%) indicating awareness of the correct procedure for collecting cervical smears. However, knowledge alone does not guarantee effective practice, highlighting the need for ongoing training within the cervical cancer screening programme. Figure 1 reveals that approximately 93% of professional nurses indicated that smears are kept at room temperature, placed in slide holders or zip bags, and collected daily.

Table 2 above presents the study findings regarding the practices of health professionals in cervical cancer screening techniques, with responses measured using a five-point Likert scale. The respondents were knowledgeable about the principles and practices of performing a Pap smear, the majority (68.5%) strongly agreed, and 21.5% agreed, respectively, that the patients should not use vaginal medical, vaginal contraceptives, lubricants, or douches for 48 hours before their Pap smear appointment. Fifty-six percent (56.2%) of the respondents attested that sexual intercourse is not recommended the night before the examination. A considerable percentage of disagreement (D = 19.2%; SD = 10.8%) is of concern to health professionals who have completed a three- or 4-year programme with clinical experience. While there was a relatively high response rate, indicating that excess blood, mucus, and inflammatory exudate could be blotted away using a gauze pad, this positive practice was not widely confirmed. Only a minority (A = 16.2%; SD = 68.5%) attested that the Pap test should follow other procedures such as cultures, tissue sampling, or the application of acetic acid. Most respondents are familiar with the practice of using cytobrushes and spatulas to collect endocervical specimens for testing. However, a concern was noted with a considerable percentage of those who disagreed (13.8% and 10.8%, respectively) about using the mentioned equipment.

**Table 2.** Screening Techniques for Cervical Cancer Screening

Statement	Disagree		Strongly disagree		Agree		Strongly agree	
	n	%	n	%	n	%	n	%
The patient should not schedule a Pap smear appointment during her menstrual periods.	27	20.8	43	33.1	19	14.6	41	31.5
The preferred time for the examination is 2 weeks after the first day of the last menstrual period.	18	13.8	5	3.8	25	19.2	82	63.1
The patient should be instructed not to use vaginal medication, vaginal contraceptives, lubricants, or douches for 48 h before her appointment.	10	7.7	3	2.3	28	21.5	89	68.5
Intercourse is not recommended the night before the examination.	25	19.2	14	10.8	18	13.8	73	56.2
The physician should not use lubricant during his examination before collecting the sample.	14	10.8	11	85	29	22.3	76	58.5
Excess blood, mucus, or inflammatory exudate may be gently blotted away with a gauze pad.	18	13.8	5	3.8	20	15.4	87	66.9
Do not scrape or wash this material away, since such actions may adversely affect the subsequent cellular sample.	13	10	6	4.6	26	20	85	65.4
The pap test should always be performed before other tests, such as cultures, tissue sampling, or the application of acetic acid.	14	10	6	4.6	26	20	85	65.4
The endocervical specimen is collected using a cytobrush, which is rotated 360° within the canal.	18	13.8	7	5.4	18	13.8	87	66.9
Ecto cervical specimen is collected with a spatula using a 360-degree rotation inside the os.	14	10.8	9	6.9	20	15.4	87	66.9
Scrape the endocervix with a spatula and spread the material rapidly onto the upper end of the slide.	17	13.1	4	3.1	20	15.4	89	68.5
Immediately spray fix by thinly soaking the cellular sample while holding the spray fixative container 6-8 inches above the slides; allow the spray fixative to evaporate slowly.	12	9.2	10	7.7	19	14.6	89	68.5
Quickly roll the endocervical brush through the ectocervical material to the end of the slides.	14	10.8	6	4.6	25	19.2	85	65.4

Regarding storage and dispatch, the majority alluded to these practices, with an average of over 70% agreeing, and the average of those who disagreed was below 10%, which is a slight concern. The findings contradict qualitative findings where other participants indicated that they are dried in the sun, and the laboratory does not collect them daily; they would come once a week or once every 2 weeks.

#### Sub-Category 1.2: Poor Filling/Completion of Cytology Form

Poor filling of the cytology forms for clients contributes to the inadequacy rate for Pap smear results, which delays early diagnosis, as failure to locate the area impacts screening. Participants confirmed this study’s findings, as indicated by the following responses:

The participant shared, *“Under filling in of the form, we have the cytology form, which is the N2 form, and on the cytology form there is an area which a clinician should complete indicating the site of the collection, the area direct the laboratory technicians to know where the specimen was collected for example if the specimen was collected from the cervix, endo cervix and expect to see endocervical cells, so when we look under the microscope when we see those cells we will say the specimen is adequate and representative of the site that the clinician says they have collected* from.” [L, Male, 39 years]

Furthermore, the participant added, “*If the form is incomplete or lacks information, the laboratory technician will assume that it is a cervical smear because 90% of the specimens that we receive for pap’ smears are cervical smears, so they will comment to say the specimen was satisfactory for evaluation with no endocervical component on the results based on the origin of the specimen. It means they will falsely contribute to inadequacy smear, we will declare them as inadequate smears only to find that they were not collected from the cervical area*.*”* [L, Male, 39 years]

Proper recording is essential for identifying the collection site, as fluids are collected from different body cavities. Adequate identification of the sample and site can accelerate an appropriate diagnosis.

#### Sub-Category 1.3: Lack of Skill in Identifying the Transformation Zone

Failure to identify the transformation zone during Pap smear collection will result in smears with no endocervical component, leading to false negative results and delaying early detection.

The lab technician shared, “*I think it is due to nurses not locating the cervix or seeing the cervix; they collect the smear anywhere where there is no endocervical component.”* [L, Male, 52 years]

Another added, *“Yes, I think it will help me do the correct thing; like I said before, I had the challenge of not seeing the cervix below. I assume there are many things we need to know about the collection of Pap smears, such as cervical screening, for example, when it is done after the menstrual period, and what to consider beforehand. I think there are those things we don’t know. The only thing I know is to insert the speculum and do Pap smear, when and how and what to consider, I don’t know, I think it will be useful, we need to be trained, mmh.”* [N5, Female, 35 years].

Another shared, “*I think I have covered most of the things; smear collectors must bear in mind that the cervix is round so it's like a clock so they need to keep a firm collection throughout when collecting and they must do it once and do 360 degrees because a lesion can be anywhere from the cervix*.*”* [N15, Female, 39 years]

The results revealed that most health care professionals were incompetent in performing cervical cancer screening. Incorporating cervical cancer screening training into the basic training of health care professionals could enhance their skills in cervical cancer screening.

#### Sub-Category 1.4: Improper Technique of Drying the Slide

Smears must be fixed immediately before they dry. A 95% solution of ethyl alcohol or spray fixative is used to fix the slide, preventing bacteria from washing away during the staining steps.

Participants indicated the following:

A professional nurse added, *“So if the clinician does not fix those cells properly, using an incorrect technique, either are too close or too far, so the preservative is not preserving the cells, and those cells will degenerate; then, for the storage of these slides to dry if you put them on the window seal at direct sunlight, the cells will become degenerate so you have to place them at room temperature.”* [N15, Female, 39 years]

She added, *“So, degenerated cells either dry, die, or break under the microscope; we won’t be able to identify them as endocervical cells and will contribute to an inadequate smear due to improper fixation.”*

The participant further added:

“*Ok, I think in terms of centimetres, I am not sure, but the literature says an elbow length, so I am not sure how many centimetres it’s an elbow length, but if you spray too close, normally the fixative becomes watery, and then it washes the slide off so it should not be done like that, should depend on the width when you spray the mist come so that mist is the one that should fall on to the cells to preserve them not the water part when the spray is near the object is too close becomes watery, it should not be watery it should be sufficient*.*”* [N15, Female, 39 years]

The requisition form typically specifies the mode of slide preservation, as the steps for processing the smear in the laboratory vary depending on the mode of fixation. This is important in ensuring that the adequacy of smears is maintained.

#### Sub-Category 1.5: Failure to Meet the Set Target for the National 70% Adequacy Rate

Monitoring and evaluation of the programme are necessary to meet the national set targets and to ensure compliance and standards, allowing for corrective measures to be taken when required.

Participants shared, *“I think that is why the laboratory service employed cytology coordinators to work in relationship with the Department of Health to improve the adequacy rate in our Primary health facilities, to capacitate and empower us to understand that is not for collecting data.”*

Another added*, “Eeh, it is to say let’s screen our women properly, so the expectation is for the NHLS together with the Department of Health to work together and improve, and go back to those facilities that are not doing well and empower them. I am also happy that you are doing this study so that this empowerment capacitation can start when nurses or clinicians are at the training level, and this can be emphasised*.*”* [N15, Female, 39 years]

Another shared, “*Ee, I can say inadequacy means that we are delaying detecting the abnormalities from our clients by not taking the correct required smear specimen. So, delaying detecting it means we are delaying the diagnosis, even delaying the treatment, so it means it will take longer for them to get the treatment*.*”* [N14, Female, 48 years]

Improving specimen quality by ensuring that health care professionals are educated on the importance of acquiring high-quality specimens through regular in-service training is crucial.

## Discussion of Findings and Literature Review

The study explored and described health professionals’ knowledge, skills, and practices regarding cervical cancer screening to ascertain the reasons for the inadequacy of cervical smears. The findings revealed that professional nurses lacked adequate knowledge of the skills required for collecting, labelling, and storing Pap smears before dispatch. Furthermore, the in-service training provided was not well coordinated and structured, and other professional nurses were not keen on doing the screening for cervical cancer. Most participants were reluctant to undergo a Pap smear screening because they were not trained or lacked confidence.

Attitudes and knowledge of nurses towards cervical cancer screening are key factors in determining the success of the programme. Rahman and Kar^23^ concur that negative attitudes towards and inaccurate knowledge of cervical cancer and screening methods among health care providers, especially among nurses, can pose substantial barriers to cervical control programmes. The majority (82.3%) understood the theory and procedure for collecting cervical smears; however, understanding the issue does not relate to practice, which suggests a need for ongoing training in the cervical cancer screening programme. Ngambi and Ramathuba^24^ also highlighted that knowledge of the screening did not translate to practice, as most participants in the study did not engage with the cervical screening programme, indicating incompetence due to inadequate developmental training programmes by district facilitators.

Moreover, another study conducted in South Africa found primary care nurses to have inadequate knowledge of cervical cancer screening.^25^ Involvement in cervical cancer screening promotes practice, and practice translates to competency. Lilliecreutz et al^26^ indicated that medical students who were capacitated through a student-led clinic cervical cancer screening (SLC-CCS) programme had the opportunity to perform pelvic examinations and collect Pap smears, which developed their professional skills, and the quality of their smears was comparable to those taken by healthcare staff when assessed.

During monitoring and evaluation meetings at the districts, inadequate smears were a concern. However, the findings highlighted that professional nurses are not accurately completing patients’ cytology forms, with most information missing, such as the clinical history, which is crucial for assessing the smears. The incompleteness of the cytology forms for clients contributes to the inadequacy rate for Pap smear results, which delays early diagnosis, as failure to locate the area where the specimen was taken impacts screening. Mubarak^27^ indicates that Quality Control (QC) in histopathology is defined as the generation of timely, accurate, and complete reports, which is best achieved in the 3 phases (pre-analytic, analytic, and post-analytic) of the laboratory testing cycles. It involves specimen collection, specimen fixation, specimen transportation, specimen/patient identification, specimen accessioning, and verification of the adequacy of the clinical history. The author further contends that quality indicators or quality monitors should be in place to monitor, identify, and evaluate performance issues throughout the crucial phases of the pre-analytical, analytical, and post-analytical processes to ensure optimal health outcomes.^27^ This suggests that all stakeholders should coordinate and make concerted efforts to achieve and maintain high-quality services through the quality monitoring and evaluation of cervical cancer programmes, thereby contributing to improved patient health outcomes.

Kamal^28^ indicates that the requisition form must specify the site of collection and mode of preservation of the slide, as the steps in processing the smear in the laboratory vary depending on the mode of fixation. Furthermore, Gurina and Simms^29^ indicate that histology examination depends on the investigation. The interpretation of the histology slide, combined with a patient’s medical history, can significantly impact the treatment course and prognosis. Therefore, providing accurate information on the type of specimen, collection site, fixation method used, and patient profile on the form contributes to the accuracy of the analysis.

The findings indicate that the technique of the fixation of the smears was not well practiced, as some professional nurses were not aware that the ethanol spray should be held 5 cm away when spraying because if it is direct, it may damage or sweep away the smear, and placing of the smear under direct sunlight is an incorrect technique. Kamal^28^ indicates that the conventional smear should be fixed immediately with absolute alcohol. Delays in fixation cause air-drying artefacts, and morphological details will be clouded. For spray fixing, the spray should be kept at a 45° angle and 6 inches from the slide. This technique was highlighted by participants as another factor that contributed to health facilities failing to meet the required target of 70% and receiving reports of inadequacy.

Bussolati^30^ indicates that the fixation step is the most critical factor in all histopathological procedures. It ensures that the structural and molecular components are fixed as they were in their living conditions. Gurina and Simms^29^ also concur that the fixation step is vital to the rest of the histologic staining procedure because it preserves the chemical composition of the tissue. The routine practice is to fix the slides immediately in 95% ethanol and send them to the laboratory for staining and evaluation by a cytopathologist. Unfortunately, laboratories often receive improper fixation due to inadequate training and excessive nurse workloads, resulting in repeated smears and delayed results.^28,31^

The study’s findings indicated that they did not meet the 70% target set by the National Health Laboratory and the Limpopo Department of Health. This target is crucial for improving the cervical cancer programme by ensuring that women are appropriately screened. Achieving this requires high-quality smears to support accurate diagnosis, enable early treatment, and ultimately improve health outcomes. However, professional nurses indicated that they were unable to locate the transformation zone. Failure to locate the transformation zone leads to false negative results because the smears are not collected at the correct site; they may be from the vaginal vault. Kamal^28^ indicates that most of the precancerous and cancerous changes occur within the transformation zone and at the Squamo Columnar Junction (SCJ), as regenerative changes in this region make it prone not only to infections but also to the entry of the human papillomavirus. Ndifon et al^32,33^ concur that most high-grade precursor lesions arise within the transformative zone or endocervical cells, which is usually considered satisfactory for evaluation. Mintzer^34,35^ indicates that smears are adequately assessed when samples from the endocervix, transformation zone, and ectocervix are present. If not, the cytopathologist must request a repeat specimen, which inflate the cost of health services.

Understanding the anatomy and physiology of the uterus is vital because the way the cervix feels to the touch will change throughout the menstrual cycle.^36^ These changes occur in response to hormonal fluctuations that facilitate the process of ovulation. Around ovulation, the cervix is as soft as the ear lobe, slightly open, and may be positioned high up in your abdomen. Other times, it’s firmer, like the tip of a nose, tightly closed and may be positioned lower down in the abdomen. Fonteyn^37^ indicates that most false negative cervical cytology results are attributable to either poor patient conditions at the time the cervical cancer specimen is collected or the way it was collected. Furthermore, Autier et al^38^ indicate that the transformation zone is located on the exocervix in 94% of women younger than 25 years old; as age increases, the proportion of women with a transformation zone situated on the exocervix decreases after 64 years. Parity is a known risk factor for cervical cancer, suggesting that with increasing numbers of live births, the transformation zone is directly exposed for more extended periods to external agents involved in dysplastic lesions.

South Africa’s health system is predominantly nurse-based. It requires nurses to be competent upon qualification to meet community health needs, and ongoing capacity development is necessary to maintain current skills. Poor training and education of health care professionals on cervical cancer screening can result in improperly collected samples, leading to false negative results and missed opportunities for early intervention. The incompetence of staff leads to inaccurate diagnoses, missed opportunities for early detection, delayed treatment, and, ultimately, a higher risk of cancer progression and mortality. Thus, the lack of structural and human resource challenges has been highlighted as a deterrent to cervical cancer screening. A lack of skills and/or capacity development, such as continuous nursing training and education programmes, as well as nursing resources and shortages of nurses, undermines and weakens nurses’ ability to improve health outcomes and health system performance.^39^ Petersen et al^40,41^ also highlighted significant gaps in cervical cancer screening services in the health systems of LMICs, ranging from a lack of high-level elements such as policies and guidelines, poor referral systems, limited points of service, and inadequate human and material resources. At the same time, Munthali et al^42^ indicate that the situation may be fueled by staff overload and challenging, constrained conditions.

Poor or uncoordinated capacity development strategies lead to a reduced quality of care, increased errors, burnout, and a decline in overall health system performance. These challenges can impact patient outcomes, increase health care costs, and hinder health systems’ ability to respond effectively to health crises. Challenges that hinder capacity-building initiatives among health care personnel in developing and LMICs include insufficient funds, inadequate infrastructure, poor leadership, and governance.^39^

The incidence of cervical cancer can be decreased by ensuring the availability of appropriately trained Pap smear providers to undertake cervical screening at PHC facilities. A Canadian study investigated whether a nurse practitioner could collect adequate Papanicolaou smear samples from the transformation zone of the cervix. The findings revealed that all smears collected by the nurse practitioner showed endocervical or metaplastic cells, indicating that nurse practitioners, if trained, can collect adequate Pap smears.^43^ Furthermore, Kottke and Trapp^44^ conducted a study in India to evaluate whether Pap test specimens collected by untrained nurses were of comparable quality to those collected by physicians, nurse practitioners, and physician assistants, were of comparable quality. The study found that nurses who were not advanced practitioners were able to collect high-quality Pap test specimens after just 1 week of training.

Mehta and Agarwal^45^ indicate that the policy prioritises the health workforce’s efficiency and competencies in screening, diagnosing, and managing precancerous cervical lesions. A capacity development programme for professional nurses should focus on strengthening individual and organisational skills through training, mentorship, and resource allocation, providing guidance and support. A clear and comprehensive plan outlining the programme’s goals, objectives, activities, timelines, and budget should be in place and collaboratively developed with concerned stakeholders.

Strategies such as in-service training, mentorship programmes, and e-learning through webinars may be helpful. The programme’s progress and impact on health outcomes and workforce capacity can be monitored and evaluated through skills assessments, performance reviews, self-evaluations, and behavioural interviews. The profession also requires continuous capacity development and provides credits for attending to ensure competency and keeping abreast with new developments and upskilling. A study conducted in Ukraine also found that capacity building for health professionals performing interprofessional management of non-communicable diseases in primary care improved their knowledge, skills, and ability to detect and assess the risk and risk factors of non-communicable diseases after training.^46^ Matus et al^47^ suggest that capacity building strategies should be interlinked and interdependent and should be implemented as part of an integrated ‘whole of system’ approach, with commitment and support from all levels of leadership and management.

Furthermore, Asah and Kaasbøll^41^ strongly recommend that educational curricula for health personnel include eHealth skills training, highlighting the importance of incorporating e-health, particularly in areas with high mobile service penetration, to aid health care efforts in regions with limited resources, as most residents have smartphones. The district coordinators, laboratory technicians, and managers must collaborate and ensure regular training programmes to improve the cervical cancer programme. The study findings have shown that integrating knowledge and awareness programmes with educational interventions of cervical cancer screening will go a long way in early detection, reducing mortality and morbidity.

## Limitations

The self-report may introduce a degree of bias and subjectivity, as it relies on individuals who voluntarily agree to participate in the study, potentially having specific interests or concerns related to the topic. Additionally, the convenience sampling process may impact the generalisability of the findings. The limited sample of laboratory technicians may have affected the external validity and, thus, generalisation to the broader population. Another limitation was the contradictory findings between the qualitative and quantitative strands of the study. The results highlighted nurses’ knowledge of Pap smear taking, but the qualitative strand indicated challenges in collecting smears. The reason is that nurses may have theoretical knowledge but not the skills to perform the task.

## Implications for Practice

Health professionals caring for women in developing countries should be adequately trained and continuously supported through monitoring and evaluation by district coordinators’ and histopathologists’ quality assurance teams in providing regular feedback to ensure individuals and PHC facility staff improve their screening skills and performance.

## Conclusion

There is a need for a coordinated approach for stakeholders involved in cervical cancer screening programmes in order to achieve and maintain the quality of cervical smear services, particularly in developing countries. There is a need to build capacity among health care professionals to enhance their skills and competencies in smear collection, thereby achieving optimal patient care and health outcomes.

## Data Availability

All data supporting this manuscript have been made available. All data transcripts coded and analysed during this study are included in this article.*
